# Fusing the spatial structure of electroencephalogram channels can increase the individualization of the functional connectivity network

**DOI:** 10.3389/fncom.2023.1263710

**Published:** 2023-10-31

**Authors:** Ming Li, Jun Yang, Wenli Tian, Xiangyu Ju

**Affiliations:** College of Intelligence Science and Technology, National University of Defense Technology, Changsha, China

**Keywords:** virtual node, EEG, individual difference, graph convolution neural network, functional connectivity

## Abstract

An electroencephalogram (EEG) functional connectivity (FC) network is individualized and plays a significant role in EEG-based person identification. Traditional FC networks are constructed by statistical dependence and correlation between EEG channels, without considering the spatial relationships between the channels. The individual identification algorithm based on traditional FC networks is sensitive to the integrity of channels and crucially relies on signal preprocessing; therefore, finding a new presentation for FC networks may help increase the performance of the identification algorithms. EEG signals are smooth across space owing to the volume conduction effect. Considering such spatial relationships among channels can provide a more accurate representation of FC networks. In this study, we propose an EEG FC network with virtual nodes that combines the spatial relationships and functional connectivity of channels. The comparison results for individual identification show that the novel EEG network is more individualized and achieves an accuracy of 98.64% for data without preprocessing. Furthermore, our algorithm is more robust in reducing the number of channels and can perform well even when a large area of channels is removed.

## 1. Introduction

Electroencephalogram (EEG) records the electrical activity in the brain, usually along the scalp surface (Jalaly Bidgoly et al., 2020). Individual differences in EEG, which result from specific patterns of individual brain activity, are mainly determined by the genetic and developmental environment (van Beijsterveldt and Boomsma, 1994). Taking advantage of the significant individual differences, EEG has been used as a more appropriate biometric than traditional methods such as fingerprints or faces (Gui et al., 2019).

In previous studies, univariate features such as the coefficients of auto-regression (AR) (Campisi and Rocca, 2014; Rocca et al., 2014; Keshishzadeh et al., 2016; Bhateja et al., 2019), power spectral density (PSD) (Palaniappan and Mandic, 2007; Ashby et al., 2011; Pham et al., 2015; Di et al., 2019), and wavelet transform (WT) (Kumari and Vaish, 2015; Kaur et al., 2017; Alyasseri et al., 2018; Yang et al., 2018) have been widely used to represent individual differences in EEG. As they are obtained by calculating signals from each electrode, univariate features are sensitive to changes in EEG amplitudes (Liu et al., 2023), which may amplify the intra-person variation. Bivariate features such as the connectivity between channels can effectively be less sensitive to amplitude interference owing to inevitably physiological or psychological factors (Zhang et al., 2021, 2022).

Functional connectivity (FC) is the most commonly used bivariate feature that captures statistical dependence or correlations between EEG channels (Bastos and Schoffelen, 2016). Ashenaei et al. found that using time-frequency FC metrics as a brain connectivity matrix can extract more discriminative features (Ashenaei et al., 2022). Chang et al. (2020) proposed a new feature extraction method based on a directed FC network and found it to be efficient for EEG-based person identification. Wang et al. (2019a). represented EEG signals as graphs based on within-frequency and cross-frequency FC estimates. The results showed that this was a more robust biometric trait than directly using the univariate features.

Traditional EEG FC networks largely rely on the functional connectivity between two channels, and recent studies have reported the limitations of such FC networks (Hassan and Wendling, 2018). Fusing other information such as the spatial relationship between channels may provide a more accurate description of FC networks. According to the volume conduction effect (Brunner et al., 2016), the signals of the two adjacent electrodes were more similar. Therefore, introducing such spatial relationship between channels could provide more information for EEG FC network.

In this study, an EEG FC network with virtual nodes that fuse spatial structures was proposed. A person identification algorithm was put forwarded using the EEG network with virtual nodes as the input of a graph convolutional neural network (VN-GCN) for EEG identification. For the data without preprocessing, our method achieved 98.64% accuracy, which is a significant improvement over existing-methods. This implies that more individualized information can be represented by integrating the spatial structure of the channels. In channel reduction experiments, VN-GCN showed less sensitivity to the number of channels and can work well even when removing a large area of channels.

The remainder of this paper is organized as follows. In Section 2, we briefly present the details of the method and the proposed VN-GCN model. In Section 3, we introduce and discuss the experimental results on person identification. Section 4 summarizes the findings of this study.

## 2. Methods

### 2.1. Functional connectivity calculation

To construct the EEG FC network, a variety of functional connectivity indices can be employed to estimate the relationship and weight between nodes, such as coherence, correlation coefficient, phase-lag index, and phase-locking value (PLV). Among them, PLV is a popular method used in EEG identification tasks. PLV (Niso et al., 2013) represents the instantaneous phase difference between two channel signals and is defined as

(1) $$PLV=\left|T^{-1}\overset{T}{\underset{t=1}{\sum }}e^{i\left(\phi_{x\left(t\right)}-\phi_{y\left(t\right)}\right)}\right|$$

where *T* denotes the time point, $\phi_{x\left(t\right)}$ and $\phi_{y\left(t\right)}$ are the phase angles of signals *x* and *y*, respectively, at the *t*-th time-point. The *PLV* ranged between [0,1], where zero and one indicate the absence of phase coupling and strict phase coupling, respectively. If all the *n* electrodes are connected to each other, we must calculate *n*(*n*–1)/2 PLV values, which can be represented by a symmetric matrix of *n* × *n* size. To reduce the storage requirement and the risk of overfitting (Behrouzi and Hatzinakos, 2022), a threshold is introduced to sparse the PLV matrix. Only the PLV values which are larger than the threshold are reserved, and others are set to be zero.

### 2.2. EEG functional connectivity network with spatial structure

**Figure 1:**
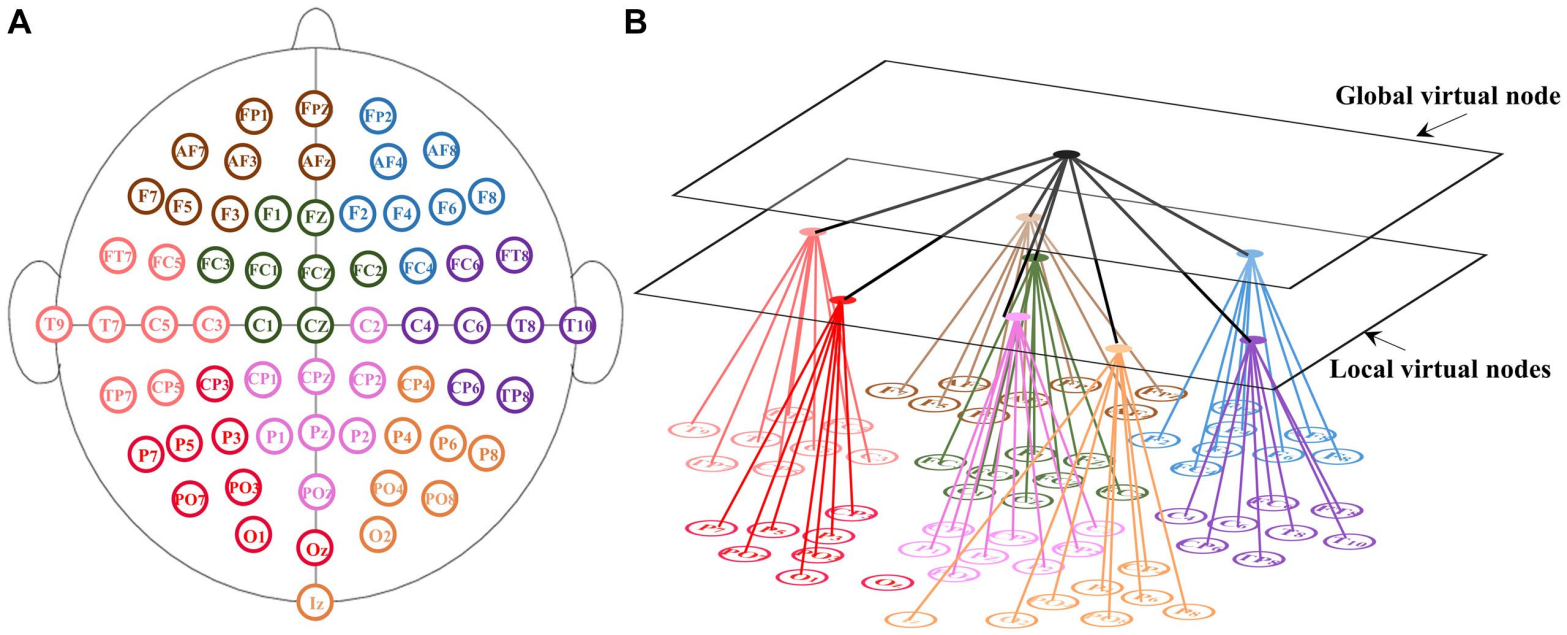
Schematic of the EEG functional connectivity network with virtual nodes. **(A)** Channel distribution of the 10–10 international system. The 64 electrodes are evenly divided into eight groups which are represented by different color. **(B)** For each group, one local virtual node was set to connect channels in the group and a global virtual node was set to connect all local virtual nodes.

Contrary to most traditional EEG FC networks, the influence of the spatial structure of channels on signal conduction patterns was considered. By employing virtual nodes, the channel information was spatially integrated. The structure of the proposed EEG network is shown in Figure 1.

The 10–10 international system is an internationally recognized method to describe and apply the location of scalp electrodes. The channel distribution of the 10–10 international system is shown in Figure 1A. It defines the spatial relationship of channels. Our FC network consists of 64 real nodes, eight local virtual nodes and one global virtual node. The 64 real nodes correspond to 64 electrodes and were divided into eight groups of equal numbers (Figure 1A). The real nodes connect to each other with weights determined by the functional connectivity. Each virtual node corresponds to one group and connects all real nodes of the corresponding group. The global-level virtual node connects all local virtual nodes. Taking the 10–10 international system as an example, the size of the new adjacency matrix is $A\in R^{73\times 73}$ and the size of the new node strength matrix is $X\in R^{73\times N}$ (*N* is the dimension of the node feature).

### 2.3. Graph convolutional neural network

A graph consisting of *n* nodes can be defined as $G=\left(V,E\right)$ , where *V* denotes the set of vertices $v_{i}\in V$ and *E* denotes the set of edges $e_{ij}\in E$ , where $i,j\in \left\{1,2,3,\cdots ,n\right\}$ . The connection relationship between vertices can be represented by an adjacency matrix $a_{ij}\in A\in R^{n\times n}$ , which indicates the connection weight between *i* and *j*. In this work, the adjacency matrix ***A*** is defined as the sparse FC matrix with virtual nodes mentioned above. Matrix $X={\left[x_{1},x_{2},\cdots ,x_{n}\right]}^{T}$ can represent the feature of all nodes. As we mainly concentrated on the connection patterns of channels, the feature of all nodes can be simply put as an all-1 matrix.

Let *L* denotes the Laplacian matrix of graph *G*, defined as:

(2) L=D−A

where $D\in R^{n\times n}$ is the diagonal degree matrix, which is calculated as:

(3) $$D_{ii}=\underset{j}{\sum }A_{ij}$$

The normalized Laplacian matrix ${\tilde{L}}_{sym}$ (Kipf and Welling, 2017) is usually used for graph convolution. Based on the renormalization technique, ${\overset{\tilde{}}{L}}_{sym}$ can be obtained by transforming the adjacency matrix *A*, which is defined as

(4) $${\tilde{L}}_{sym}={\tilde{D}}^{-1/2}\tilde{A}{\tilde{D}}^{-1/2}$$

where $\tilde{A}$ and $\tilde{D}$ are defined by:

(5) $$\tilde{A}=A+I$$

(6) $$\tilde{D}=\overset{n}{\underset{j=1}{\sum }}{\tilde{A}}_{ij}$$

The GCN graph convolution is realized by aggregating the features of the nodes and their adjacent nodes. The single convolutional layer *k* of GCN can be defined as

(7) $$X^{L_{k+1}}=\sigma \left({\tilde{L}}_{sym}X^{L_{k}}W^{L_{k}}+b^{L_{k}}\right)$$

where $X^{L_{k}}$ means the input of the *k*-th graph convolutional layer and $X^{L_{k+1}}$ means the output of the *k*-th graph convolutional layer. $W^{L_{k}}$ and $b^{L_{k}}$ are the weight matrix and the bias matrix of the *k*-th graph convolutional layer, σ represents the activation function.

### 2.4. VN-GCN framework for EEG identification

**Figure 2:**
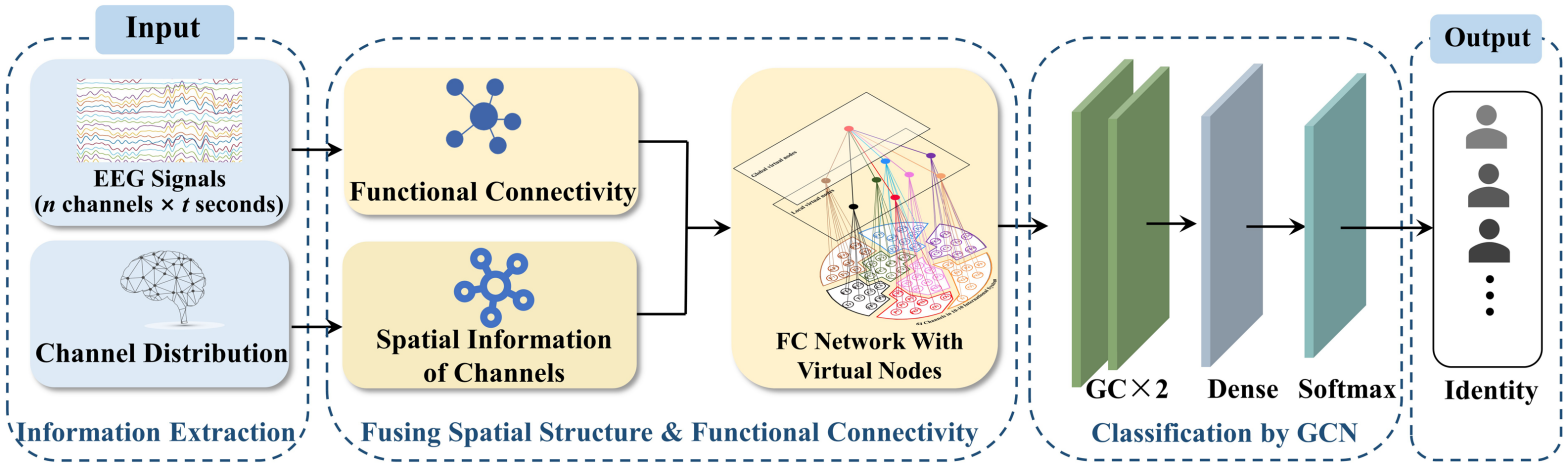
VN-GCN framework for EEG identification.

The design of the VN-GCN model, which uses the EEG FC network with virtual nodes as the input of the GCN, is shown in Figure 2. The EEG signals and distribution of channels can be obtained from the acquisition process. The original EEG network was constructed by computing the functional connectivity between the EEG signals. By combining the spatial information of the channels, an EEG FC network with virtual nodes was constructed, as shown in Section 2.2, and was set as the input of the two graph convolution layers. Subsequently, a flattened layer expanded the output matrix into one dimension, and a dense layer was used to realize mapping from the feature to label spaces. Finally, a softMax layer was used to classify the output layer. The network was trained by iterating categorical cross entropy loss.

## 3. Results and discussion

In order to evaluate the individualization of EEG FC network after adding virtual nodes, person identification experiments based on VN-GCN were designed. The raw EEG data (*n* channels × *t* seconds) from different subjects was feed to the input terminal of VN-GCN. And the task of VN-GCN is to predict the identity tags of the subjects at the output terminal (see Figure 2). The accuracy of the prediction is calculated to evaluate the individualization of FC network with virtual nodes, and compared with person identification methods which based on traditional FC network.

### 3.1. Dataset

An online dataset, the PhysioNet EEG Motor Movement/Imagery Dataset (Goldberger et al., 2000; Schalk et al., 2004), was used to evaluate the performance of the VN-GCN and other methods. This dataset consists of EEG recordings from 109 volunteers, each of which performed 14 experimental runs: two one-minute baseline runs [one with eyes open (EO) and the other with eyes closed (EC)], and three two-minute runs of each of the following four tasks (opening or closing the left or right fist, imaging opening or closing the left or right fist, opening or closing both fists or both feet, and imaging opening or closing both fists or feet). EEGs were recorded from 64 electrodes (*n* = 64) as the 10–10 international system.

To evaluate the generalizability of VN-GCN, another popular public EEG dataset, the UCI KDD EEG dataset (Zhang et al., 1995) was also used to make further experiments. The records of this dataset are obtained by 64 electrodes (*n* = 64) at a sampling rate of 256 Hz. The number of subjects was 122 including 45 normal and 77 alcoholic males.

A moving window of 1 s with a 50% overlap was used to generate the training and testing samples. Most existing studies tend to add preprocessing steps such as ICA (Winkler et al., 2014) to reduce the noise of the original EEG signals. However, in practice, different noise reduction methods should be designed based on the EEG acquisition equipment. This indicates that the model requires more computation and time. In the subsequent experiments, EEG data without preprocessing were used.

### 3.2. Model accuracy and comparison

The identification accuracy of our VN-GCN was compared with that of two of the most popular EEG identification models developed in recent years. These are Tina’s GCN model, which was proposed in 2021 (Behrouzi and Hatzinakos, 2021), and an EEG identification model based on CNN and functional connectivity, which was proposed by Wang in 2019 (Wang et al., 2019b). The structures of these three models are listed in Table 1. To evaluate the computational complexity in the training of VN-GCN and the other two model, the parameter amounts of each layer are listed in Table 2. In creating a personalized FC network, the major computational step is calculating the PLV between each pair of channels, so the computational complexity of constructing a FC network is $O\left(T\cdot n^{2}\right)$ , where *T* is the number of time points, *n* is the number of channels.

**Table 1:** Structures of Tina’s GCN, CNN-FC, and our VN-GCN model.

Model	Layer	Output
Tina’s GCN	Graph Convolution I	64 × 32
	Graph Convolution II	64 × 8
	Flatten	512 × 1
	Dense	109 × 1
	SoftMax	109 × 1
CNN-FC	Convolution I	63 × 63 × 32
	Pooling I	31 × 31 × 32
	Convolution II	30 × 30 × 32
	Pooling II	15 × 15 × 32
	Flatten	7,200 × 1
	Dense	109 × 1
	SoftMax	109 × 1
VN-GCN	Graph Convolution I	63 × 32
	Graph Convolution II	73 × 8
	Flatten	584 × 1
	Dense	109 × 1
	SoftMax	109 × 1

**Table 2:** Comparison of the parameter amount.

Model	Layer	Paraments	All
Tina’s GCN	Graph Convolution I	3.1 k	59.8 k
	Graph Convolution II	768	
	Dense	55.9 k	
CNN-FC	Convolution I	160	785.2 k
	Convolution II	160	
	Dense	784.9 k	
VN-GCN	Graph Convolution I	3.4 k	**68.0 k**
	Graph Convolution II	840	
	Dense	63.8 k	

The comparison results on the PhysioNet EEG Motor Movement/Imagery Dataset are presented in Table 3, and the results on the UCI KDD EEG dataset are given at Table 4. Any preprocessing was not conducted. A five-fold cross-validation was performed.

**Table 3:** Accuracy of the three models under resting state EO and EC.

Model	EO	EC
	ACC(%)	ACC(%)
CNN-FC	89.72 ± 0.57	86.33 ± 0.68
Tina’s GCN	95.15 ± 0.12	92.19 ± 0.14
VN-GCN	**98.64 ± 0.23**	**92.96 ± 1.50**

**Table 4:** Accuracy of the three models on the UCI KDD EEG dataset.

Model	Alcohol	Normal
	ACC(%)	ACC(%)
CNN-FC	88.27 ± 3.91	84.95 ± 2.87
Tina’s GCN	89.45 ± 3.54	85.24 ± 2.41
VN-GCN	**89.79 ± 3.32**	**86.09 ± 3.34**

Clearly from the results in Table 3, the accuracy achieved by the VN-GCN model was 8.66 and 2.93% higher than that of the CNN-FC and Tina’s GCN models, respectively, when using EEG data in the resting-state EO. For the EC, the values were 6.63 and 0.77%, respectively. This demonstrates that VN-GCN can achieve higher accuracy with un-preprocessed data and fuse the spatial structure of channels and functional connectivity through virtual nodes. Comparing the accuracy in the resting-state EO and EC, it can be found that the accuracy at EC was much lower than that in EO. One possible reason for this is that people tend to have better control over other brain activities during EO, whereas the brain is more active and uncontrollable during EC. The result on the UCI KDD EEG dataset also shown that our VN-GCN model could achieve the best results.

### 3.3. Effects of channel reduction

**Figure 3:**
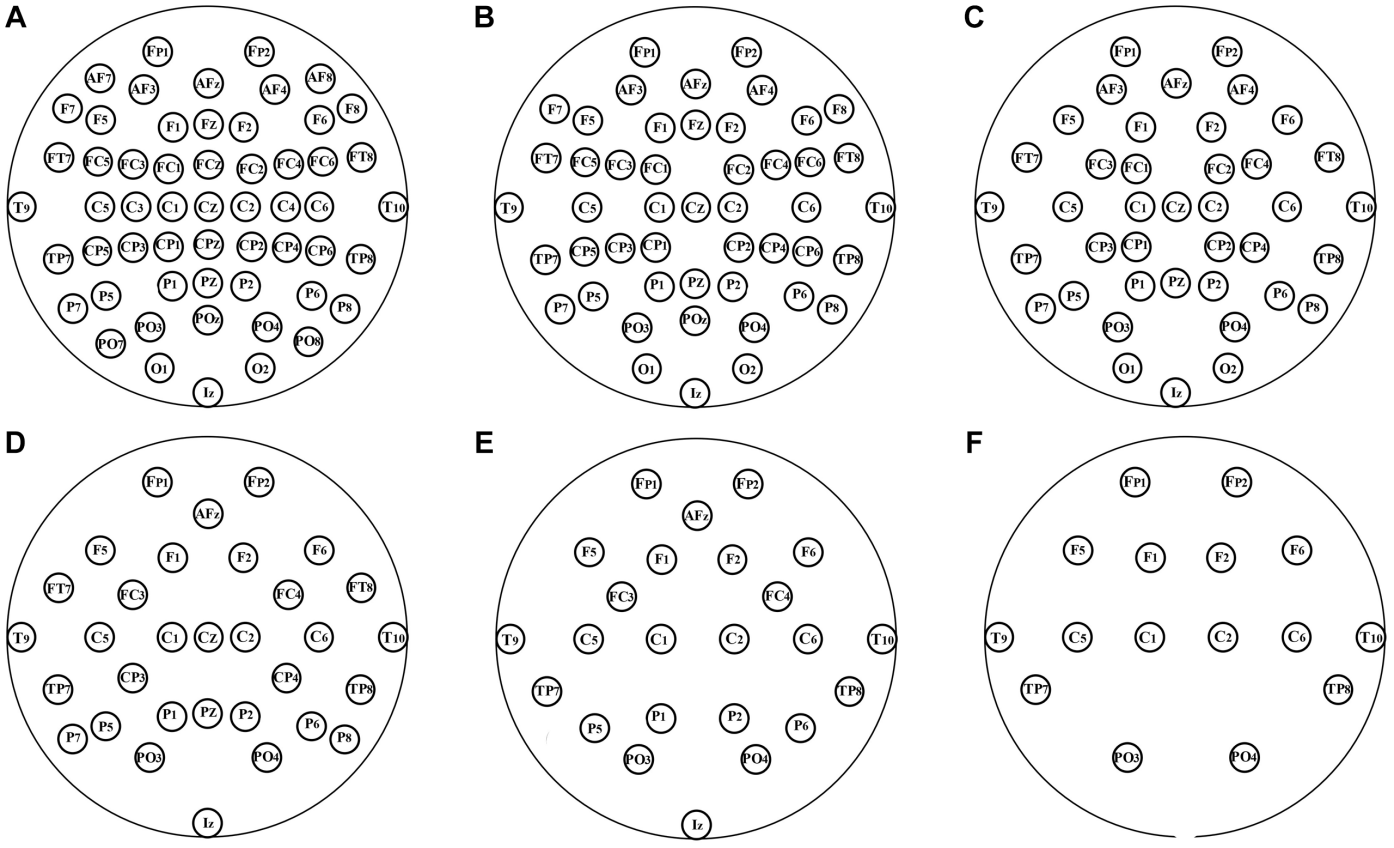
Layout of channels after uniformly reduction. **(A–F)**: 56, 48, 40, 32, 24, and 16 channels were, respectively, retained.

In the aforementioned experiment, 64-channel EEGs were used. In theory, more channels of EEG signals indicate that the characterization of brain connections is more detailed, and the EEG FC network is more individualized. However, higher costs are incurred in both the data acquisition and analysis stages when there are more channels. Channel-reduction experiments were conducted to provide support for the application of the technology in the case of fewer channels. The original distribution of the 64 channels was uniformly reduced to 8, 16, 24, 32, 40, and 48 channels, as shown in Figure 3.

**Figure 4:**
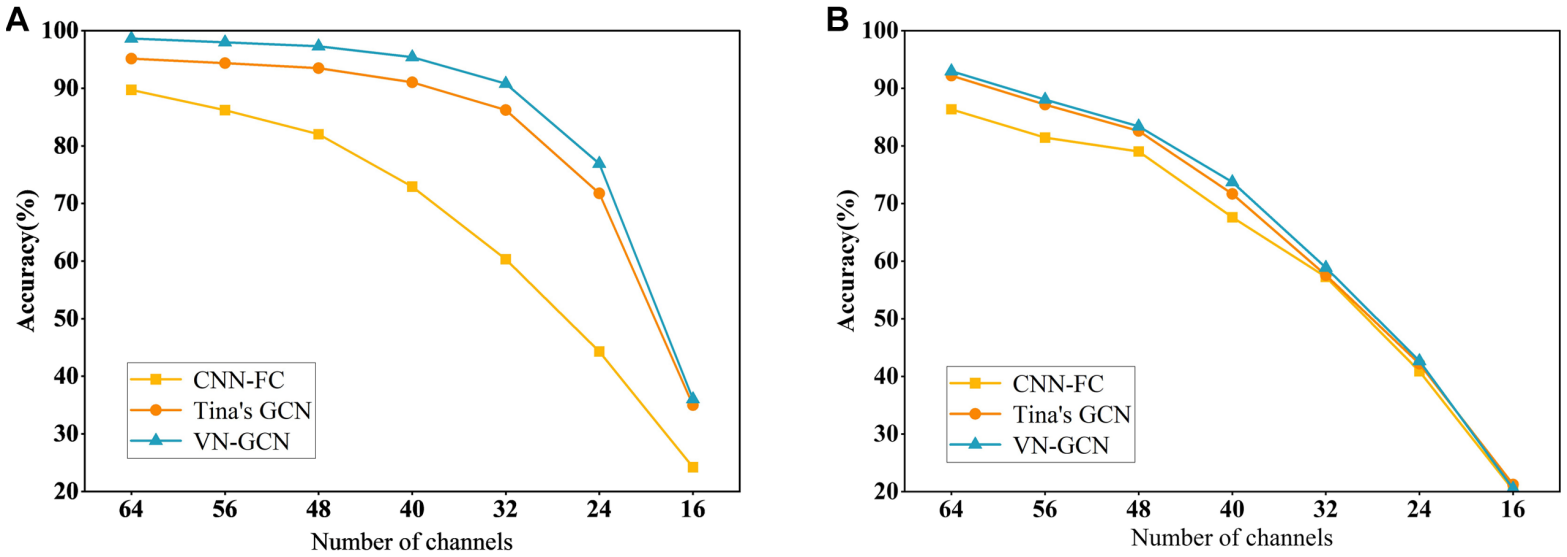
Comparison of classification accuracy of three models after uniformly reducing the channels. **(A)** In resting state EO and **(B)** in resting state EC.

The experiment was conducted when the three models had the same structure described in Section 3.2, and the same functional connectivity (PLV) was used. The results are summarized in Table 5 and compared in Figure 4.

**Table 5:** Classification accuracy of three models after uniformly reducing the channels.

Number of channels	Model	EO	EC
		ACC(%)	ACC(%)
56	CNN-FC	86.20	81.44
	Tina’s GCN	94.37	87.17
	**VN-GCN**	**97.96**	**88.03**
48	CNN-FC	82.02	79.03
	Tina’s GCN	93.49	82.60
	**VN-GCN**	**97.28**	**83.40**
40	CNN-FC	72.91	67.61
	Tina’s GCN	91.03	71.66
	**VN-GCN**	**95.41**	**73.71**
32	CNN-FC	60.31	57.26
	Tina’s GCN	86.22	57.55
	**VN-GCN**	**90.79**	**58.85**
24	CNN-FC	44.29	40.89
	Tina’s GCN	71.77	42.20
	**VN-GCN**	**76.90**	**42.66**
16	CNN-FC	24.23	20.25
	Tina’s GCN	35.01	21.22
	**VN-GCN**	**36.95**	**20.53**

The results indicate that the VN-GCN model was more robust than Tina’s GCN and CNN-FC models. When using EEG data in resting-state EO, the accuracy of VN-GCN changed slightly and was maintained at more than 90% when the number of the removed channels was less than half. When reduced to only 16 channels, the classification accuracy of the VN-GCN model was less than 40%, which is similar to that of Tina’s GCN and CNN-FC models.

Therefore, when using EEG data with fewer channels, the number of channels should be maintained between 64 and 32 to ensure the accuracy of the model.

### 3.4. Sensitive to brain region removal

**Figure 5:**
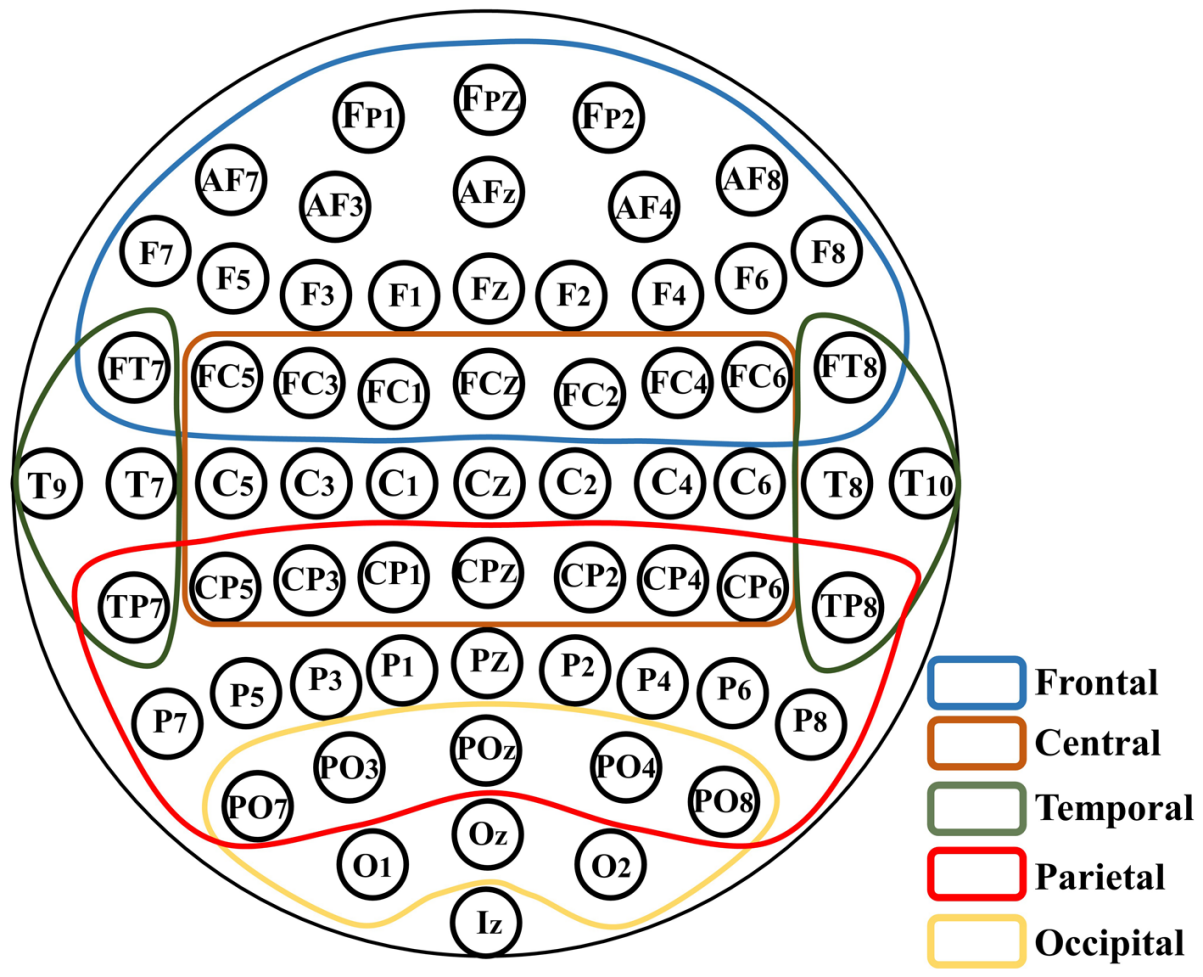
Schematic of the brain region removal. All the channels are divided into five regions which are overlapping according to their signs in the 10–10 international system.

The arrangement of EEG channels on the brain surface corresponds to specific brain regions. To explore the contribution of brain regions to individual differences, an experiment on brain region removal was conducted. Figure 5 shows the relationship between the channels and brain regions based on 10–10 international system.

**Figure 6:**
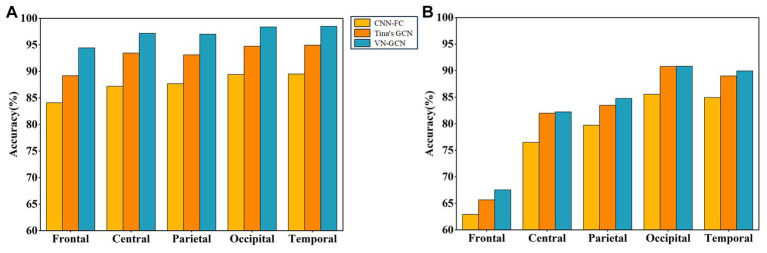
Comparison of classification accuracy of three models after brain region removal. **(A)** In resting state EO and **(B)** in resting state EC.

According to the division of brain regions given above, we successively removed the corresponding channels and compared the accuracy of VN-GCN with that of Tina’s GCN and CNN-FC models. The results are presented in Table 6 and compared in Figure 6.

**Table 6:** Classification accuracy of the three models with brain region removal.

Removed brain areas	Models	EO		EC	
		ACC(%)	Drop(%)	ACC(%)	Drop(%)
Frontal	CNN-FC	84.12	5.60	62.93	23.40
	Tina’s GCN	89.20	5.95	65.66	26.53
	**VN-GCN**	**94.44**	**4.20**	**67.55**	**25.41**
Central	CNN-FC	87.20	2.52	76.51	9.82
	Tina’s GCN	93.47	1.68	82.00	10.19
	**VN-GCN**	**97.17**	**1.47**	**82.23**	**10.73**
Parietal	CNN-FC	87.70	2.02	79.71	6.62
	Tina’s GCN	93.13	2.02	83.47	8.72
	**VN-GCN**	**97.00**	**1.64**	**84.76**	**8.20**
Occipital	CNN-FC	89.42	0.30	85.56	0.77
	Tina’s GCN	94.76	0.39	90.79	1.40
	**VN-GCN**	**98.37**	**0.27**	**90.83**	**2.12**
Temporal	CNN-FC	89.52	0.20	84.94	1.39
	Tina’s GCN	94.95	0.20	89.01	3.18
	**VN-GCN**	**98.49**	**0.15**	**89.97**	**2.99**

First, the VN-GCN had the lowest classification accuracy drop compared to the other two models when the brain regions were removed. It can also be seen that when channels are removed according to the brain regions, the change rule of the accuracy of the three models is the same. The classification accuracy decreased the most when the channels of the frontal lobe region were removed and least when the channels of the occipital and temporal lobe regions were removed. This is because the frontal lobe region has the largest number of channels. The number of channels in the parietal and central lobe regions was not significantly different from that in the frontal lobe region; however, the accuracy drop in the former was much smaller. Second, this rule seems to be amplified in resting-state EC, which means that the models are more sensitive in this state.

## 4. Conclusion

In this study, an EEG FC network that fuses the spatial structures of channels was proposed and used for EEG identification. By introducing virtual nodes that communicate with local channels, the structural information of the channels can be integrated into the FC network. The basic principle of integrating the spatial structure of channels is based on the volume conduction effect, which reflects the EEG conduction pattern. Taking an FC network with virtual nodes as the input to the graph convolutional network, the VN-GCN model was proposed for person identification tasks. The results show that, compared with traditional methods that construct an EEG network with only functional connectivity, VN-GCN achieves a higher identification accuracy. In the channel-reduction experiments, the results demonstrated that VN-GCN is more robust against channel reduction, and the result still holds when the channels of an entire brain region are removed. However, adding virtual nodes increases the scale of the FC network, which leads to larger computational complexity of the model, and this may limit the application in some cases. Besides, the local virtual nodes can only integrate the electrodes in the same group and cannot integrate electrodes in different groups, even if they are spatially adjacent. Finally, although our method can achieve the accuracy of more than 98% without preprocessing, the performance of the method in the presence of noise or artifacts needs further study.

In conclusion, by integrating the spatial structure with virtual nodes, the EEG FC network was more discriminated and robust among different people. Moreover, this is an effective method to achieve more accessible EEG-based person identification for use in real-world scenarios.

## Data availability statement

The original contributions presented in the study are included in the article/supplementary material, further inquiries can be directed to the corresponding author.

## Author contributions

ML: Writing – original draft, Writing – review & editing. JY: Writing – original draft, Writing – review & editing. WT: Writing – review & editing. XJ: Writing – review & editing.
